# A phase II study of mitomycin C, cisplatin and continuous infusion 5-fluorouracil (MCF) in the treatment of patients with carcinoma of unknown primary site

**DOI:** 10.1038/sj.bjc.6600258

**Published:** 2002-04-22

**Authors:** A G Macdonald, M C Nicolson, L M Samuel, A W Hutcheon, F Y Ahmed

**Affiliations:** ANCHOR Unit, Aberdeen Royal Infirmary, Aberdeen AB25 2ZN, UK; Northern Centre for Cancer Treatment, Newcastle General Hospital, Westgate Road, Newcastle-upon-Tyne NE1, UK

**Keywords:** MCF, adenocarcinoma, carcinoma of unknown primary site

## Abstract

Carcinoma of unknown primary site remains a common clinical diagnosis, accounting for between 5 and 10% of all cancer patients. Numerous combination chemotherapy regimens have been used in the management of carcinoma of unknown primary site, resulting in response rates of 0–48%. We present the results of a single centre phase II study of the use of the combination of mitomycin C (7 mg m^−2^ on day 1 of cycles 1, 3 and 5) cisplatin (60 mg m^−2^ on day 1) and continuous infusion 5-fluorouracil (300 mg m^−2^ daily), MCF, delivered as a 21-day cycle, in patients with carcinoma of unknown primary site. Thirty-one patients with a diagnosis of carcinoma of unknown primary site were treated in Aberdeen Royal Infirmary between 1997 and 2001 with MCF. In total, 136 cycles of MCF were delivered (median of 5 cycles per patient). Toxicity was acceptable, with 19% grade 3 or 4 neutropenia, 16% grade 3 or 4 thrombocytopenia and 13% grade 3 or 4 nausea and vomiting. No cases of neutropenic sepsis were seen and there were no treatment-related deaths, however, six patients developed thrombotic complications. The overall response rate was 27% (CR 3%; PR 23%). Median time to progression was 3.4 months (95% CI 1.1–5.6 months) and median overall survival was 7.7 months (95% CI 5.7–9.8 months). Survival at 1 year was 28%, and at 2 years, 10%. MCF is a tolerable regimen with comparable toxicity, response rates and survival data to most platinum-based combination chemotherapy regimens in use for this devastating disease.

*British Journal of Cancer* (2002) **86**, 1238–1242. DOI: 10.1038/sj/bjc/6600258 www.bjcancer.com

© 2002 Cancer Research UK

## 

The diagnosis of carcinoma of unknown primary site accounts for 5–10% of all new patients referred to oncology clinics (Greco and Hainsworth, 1992). Of these, about 60% are identified on light microscopy and immunohistochemistry as being adenocarcinomas (Hainsworth and Greco, 1993). Adenocarcinoma of unknown primary site (ACUP) is a diagnosis which generally carries a poor prognosis. Historically, the median survival for this condition has been reported as 3.1 months (Markmann, 1982), however, more recent phase II studies of combination chemotherapy have generally resulted in median survival times of over 6 months. Certain subgroups of patients have been identified with a more favourable prognosis, namely patients with neuro-endocrine tumours (Lenzi *et al*, 1997), women with lone axillary metastases (Ellerbroek *et al*, 1990) or diffuse peritoneal carcinomatosis (Strnad *et al*, 1989), and men with an elevated serum prostate-specific antigen (PSA) or with tumour that stains for PSA (Tell *et al*, 1985).

The most common metastatic sites in ACUP are lymph nodes, lung, liver and bone. Intensive investigation rarely identifies a primary site, and if successful, seldom alters treatment (Stewart *et al*, 1979). Therefore, only a limited search for the primary site is generally adopted in the absence of specific symptomatology. Tests include complete physical examination, tumour markers, stool Haemoccult test, chest radiography and abdominopelvic CT scan, as well as mammography in women. Further investigations, such as chest CT scan, endoscopy and barium studies, may be performed if the clinical scenario is suggestive of a particular primary site.

In only 15–20% of patients with ACUP does the primary site subsequently declare itself during life, however, post-mortem studies can define the primary in some 70–80% of cases (Nystrom *et al*, 1977). From such examinations, it can be seen that the commonest origins for ACUP are lung and pancreas, followed by stomach, colon and oesophagus, explaining the generally poor prognosis of these patients.

No large randomised trials of chemotherapy *vs* best supportive care have been performed, however, an analysis of the outcomes of 222 patients with hepatic metastases from ACUP by Ayoub *et al* (1998), showed that delivery of chemotherapy was associated with improved survival (hazard ratio 0.43, *P*<0.0001), after adjusting for age and number of metastatic sites. Single agent chemotherapy studies in patients with ACUP show response rates for 5-fluorouracil (5-FU) and cisplatin of 0–16% and 19% respectively (Johnson *et al*, 1964; Moertel *et al*, 1972; Schildt *et al*, 1983; Wagener *et al*, 1991). Numerous phase II studies have been performed with combination chemotherapy regimens (Anderson *et al*, 1983; Jadeja *et al*, 1983; van der Gaast *et al*, 1988; Becouarn *et al*, 1989; Treat *et al*, 1989; Hainsworth *et al*, 1997; Warner *et al*, 1998; Lofts *et al*, 1999; Briasoulis *et al*, 2000; Dowell *et al*, 2001; Greco *et al*, 2001; Guardiola *et al*, 2001), generally achieving response rates between 10 and 40%. More recently, the addition of paclitaxel, with its broad spectrum of activity, into combination regimens, has yielded response rates approaching 50% (Hainsworth *et al*, 1997; Greco *et al*, 2000). One problem with comparison of these studies is the heterogeneity of the study populations and differing stringency with which investigators attempt to exclude breast cancer, ovarian cancer and germ cell tumours as potential primary sites, i.e. those tumours which are associated with better survival and which can therefore markedly skew outcome data.

The rationale for studying MCF lies in the activity of mitomycin C in lung and gastrointestinal (G-I) cancers, of cisplatin in lung, breast, ovarian and upper G-I cancers, and of 5-FU in breast and G-I cancers, thereby producing a combination with potential activity against the main primary tumour sites responsible for ACUP. In addition, the three agents give rise to generally non-overlapping toxicities.

## MATERIALS AND METHODS

### Patient selection and investigation

Patients were eligible for the study if there was a cytologically or histologically confirmed diagnosis of carcinoma of unknown primary. Patients were required to be aged between 18 and 75, chemo-naïve, have a WHO performance status of ⩽2 and have adequate haematological, renal and liver function.

All patients were evaluated clinically by medical history and physical examination. Baseline investigations included full blood count, serum biochemistry and tumour markers (CEA and Ca125 in women; CEA and PSA in men, plus βHCG and AFP in cases of poorly differentiated carcinomas in males under 50 years). Plain chest radiographs and abdominopelvic CT scans were performed, with mammography in women. Further investigations, such as chest CT scan, pelvic ultrasound, endoscopy, barium studies and bone scintigraphy were performed dependent on the patient's symptoms or signs.

All pathology was analysed centrally. Where adenocarcinoma was diagnosed on light microscopy, immunohistochemistry was performed on the pathological specimen for CEA and PSA in men, and for CEA, Ca125 and hormone receptors in women. Where poorly differentiated tumour was diagnosed on light microscopy and immunohistochemistry, appropriate stains were used to confirm carcinoma (including cytokeratins and epithelial membrane antigen) and exclude haematological malignacies, melanoma, germ cell tumours and sarcoma (including common leucocyte antigen, CD30, S-100, βHCG, AFP and vimentin).

Patients were excluded if tumour markers, radiology and/or the clinical scenario were in keeping with primary prostatic cancer (PSA >10 ng ml^−1^ and/or bone only disease in a male), ovarian or primary peritoneal cancer (women with only peritoneal disease and Ca125 >50 U ml^−1^), or germ cell neoplasm (predominantly midline poorly differentiated tumour in a male under the age of 50, with or without elevated βHCG or AFP), or if they had nodal disease only which was localised to a single lymph node region.

### Treatment schedule

The MCF regimen was delivered every 21 days for a maximum of six cycles. Mitomycin C (7 mg m^−2^; maximum dose 14 mg) was delivered on day 1 of every alternate cycle. Cisplatin (60 mg m^−2^ with pre- and post-hydration, frusemide and mannitol) was delivered on day 1 of each cycle. 5-FU was delivered as a continuous infusion (300 mg m^−2^ day) throughout treatment, via a tunnelled catheter and portable pump. Prophylactic warfarin (1 mg daily) was given to reduce the incidence of line-associated thrombosis. Prophylactic anti-emetic therapy consisted of 8 mg dexamethasone and 8 mg ondansetron pre-treatment, and thereafter 2 mg dexamethasone t.d.s and 8 mg ondansetron b.d. for 3 days and was altered as required. Chlorhexidine mouthwash was supplied to all patients.

Prior to each cycle of therapy (whether mitomycin C was due or not), adequate haematological function (neutrophil ⩾1.0×10^9^ per litre and platelets ⩾100×10^9^ per litre) was required, otherwise chemotherapy was delayed (and the infusional 5-FU discontinued) for 1 week or until the myelosuppression had resolved. Renal function was monitored by calculating creatinine clearance prior to each cycle, to ensure a clearance of ⩾60 ml min^−1^. For values below 60 ml min^−1^, the total dose of cisplatin per cycle was reduced to the GFR value in mg, and below 40 ml/min, cisplatin was omitted.

In the event of oral mucositis or grade 2 diarrhoea, infusional 5-FU was discontinued for at least 1 week, then reinstituted at a reduced dose of 250 mg m^−2^ when the symptoms resolved. If still not tolerated, the dose was reduced further to 200 mg m^−2^.

### Toxicity

Toxicity was assessed for all cycles according to NCI Common Toxicity Criteria version 2.0, and was recorded as the worst toxicity experienced per patient.

### Response evaluations

The outcomes measured were tumour response (evaluated according to WHO response criteria), time to progression and overall survival. Response was assessed at each cycle by clinical examination, tumour markers and CXR if appropriate. CT scans were repeated after 3 and 6 cycles of chemotherapy. Although WHO response criteria were used, it was not possible to confirm responses after 1 month because of resource limitations. Time to progression and overall survival were defined as the time from the first cycle of therapy to the date of documented progression (clinical or radiological) or death, respectively.

### Statistical analyses

The response rates for previous platinum- or taxane-based regimens in carcinoma of unknown primary lie between 19 and 50% (see Table 5). Assuming a 35% response rate in a sample of 30 evaluable patients, the 95% confidence interval would be 10 to 50%.

Median time to progression and median overall survival were estimated by the Kaplan Meier method (Kaplan and Meier, 1958) using SPSS version 9.0.

## RESULTS

Thirty-one consecutive eligible patients with CUP were recruited into the study at Aberdeen Royal Infirmary between April 1997 and January 2001. The patient characteristics are listed in Table 1

**Table 1:**
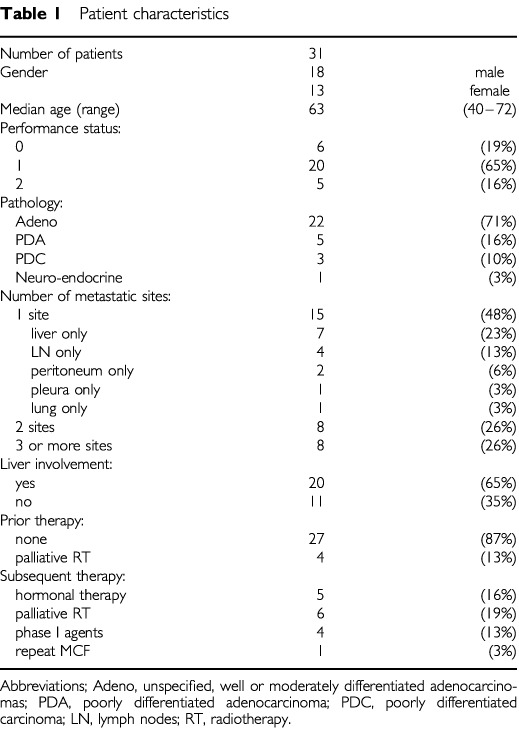
Patient characteristics

. None of the four patients with only nodal metastases had disease which was localised to a single radically-treatable subsite (with widespread retroperitoneal lymphadenopathy in three cases, and mediastinal, axillary and cervical lymphadenopathy in the fourth). The two patients with solely peritoneal disease were both investigated by specialist gynaecological oncology surgeons, and had normal or marginally-elevated serum Ca125 and adenocarcinomas that did not stain positively for Ca125. In both cases, the clinical scenario, the tumour histology and the pattern of disease found at operation were felt to make the diagnosis of ovarian or primary peritoneal carcinoma unlikely.

### Drug delivery

A total of 136 cycles of MCF chemotherapy was delivered, with a median of 5 cycles per patient (range 2–6). Twenty-two cycles were delayed, most by only 1 week. The reasons for delay were myelosuppression in 14 cases, stomatitis in four cases, and grade 3 vomiting, grade 4 constipation, grade 2 diarrhoea and unexplained jaundice in one case each.

Dose reductions were instituted for mitomycin C in two patients (due to neutropenia), for cisplatin in four patients (due to impaired renal function from the start of chemotherapy in two cases and multiple toxicities in two cases) and for 5-FU in 12 patients (for reasons of stomatitis in seven, palmar-plantar syndrome in three, and diarrhoea and neutropenia in one case each).

The delivered dose intensity for each drug was calculated by averaging the mean dose received per week for the entire treatment course for each patient, and the results are compared with the intended dose intensities in Table 2

**Table 2:**
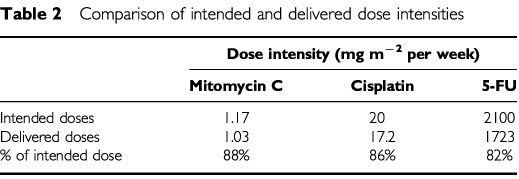
Comparison of intended and delivered dose intensities

.

### Toxicity

No treatment-related deaths were observed within the study. There were 12 emergency admissions in 11 patients. The reasons for admission were thrombotic complications in four cases, and one case each of urinary retention, rigors with no other evidence of infection, grade 3 vomiting, grade 4 thrombocytopenia and grade 4 anaemia. The remaining three admissions arose as a consequence of disease progression rather than therapy, two with bowel obstruction and one with obstructive uropathy.

All patients were assessable for toxicity and the data are summarised in Table 3

**Table 3:**
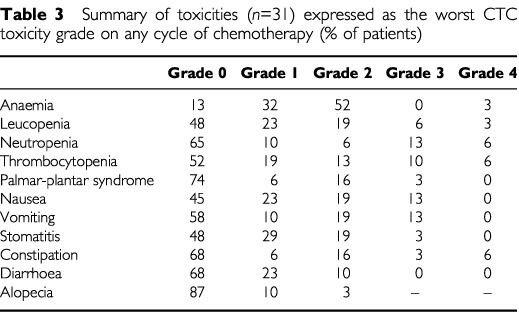
Summary of toxicities (*n*=31) expressed as the worst CTC toxicity grade on any cycle of chemotherapy (% of patients)

. Nineteen per cent of patients experienced grade 3 or 4 neutropenia (none with sepsis), 16% with grade 3 or 4 thrombocytopenia and 13% with grade 3 nausea and vomiting. No cases of haemolytic uraemic syndrome were seen.

5-FU related toxicity was common, with 48, 33 and 22% of patients experiencing grade 1 or 2 stomatitis, diarrhoea and palmar-plantar syndrome respectively, however, severe toxicity was rare.

No patients developed significant chemotherapy-related nephrotoxicity, although the calculated creatinine clearance fell by between 10 and 20% in five patients from the start to the end of chemotherapy. In no case did calculated renal function fall by more than 20%.

A total of six Hickman line complications occurred in four patients. There were three episodes of subclavian vein thrombosis, two episodes of line infection and one pneumothorax.

### Response

Thirty patients had measurable disease and were included in the response assessment. After six cycles of MCF, eight patients had responded to chemotherapy, one complete response (3%) and seven partial responses (23%), giving an overall response rate of 27% (95% CI 11–42%). In total, 63% of patients progressed during their chemotherapy. Of the 10 patients who had stable disease after three cycles, two subsequently achieved a partial response (both of whom had shown a minor response after three cycles), three maintained stable disease and five had progressed by completion of treatment. Of the eight patients who had a partial response after three cycles, two had progressed by the end of the sixth cycle; in view of the lack of a confirmatory 1 month scan, the initial responses of these two patients were not included in the overall response rate.

All eight patients who achieved a response after six cycles of chemotherapy had a histological diagnosis of adenocarcinoma (as opposed to poorly differentiated carcinoma), six with liver involvement and one each with node only and peritoneum only disease.

Table 4

**Table 4:**
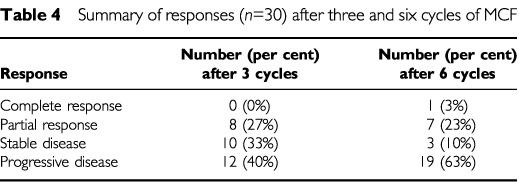
Summary of responses (*n*=30) after three and six cycles of MCF

summarises the response data.

### Survival

**Figure 1:**
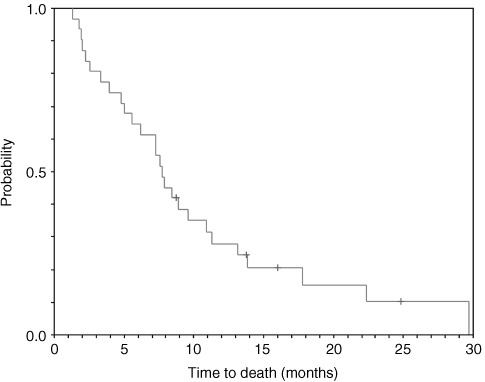
Kaplan–Meier curve of overall survival (*n*=31).

Survival data were available for all 31 patients. After a median of 25 months follow-up (range 7–53 months), the survival data are mature. Median time to progression is estimated as 3.4 months (95% CI 1.1–5.6 months) and median survival as 7.7 months (95% CI 5.7–9.8 months (Figure 1

)). Actuarial 1-year survival is 28% and 2-year survival is 10%. Meaningful multivariate analysis is precluded by the small patient population in this study, however, of the eight patients who had survived 1 year by the time of the analysis, all had a performance status of 0 or 1, seven had a histological diagnosis of adenocarcinoma (the eighth being diagnosed with a neuro-endocrine tumour) and six had disease involving the liver. One patient had solely nodal disease.

## DISCUSSION

The MCF regimen was found to be generally well tolerated in patients with carcinoma of unknown primary, with grade 3 or 4 toxicity rates which are very similar to those reported for the same regimen when used in gastric carcinoma (Ross *et al*, 1999). Twelve patients required dose reductions of 5-FU, three of whom required a second dose reduction, mainly for reasons of stomatitis and palmar-plantar syndrome. This level of toxicity suggests that a daily dose of 250 mg m^−2^ of 5-FU may be preferable as a starting dose.

A response rate of 27% was seen. Of the eight patients who had stable disease with no evidence of even a minor response after three cycles of MCF, five had progressed by the sixth cycle and only three maintained stability, raising the suggestion that in those patients without any demonstrable reduction in tumour size after three cycles, MCF should be discontinued.

The 27% response rate and 7.7 month median survival observed with MCF are broadly similar to other cisplatin-based regimens (see Table 5

**Table 5:**
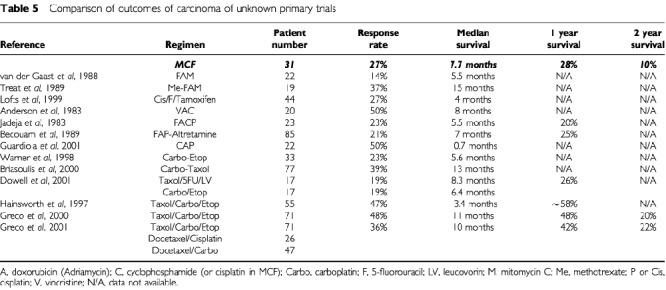
Comparison of outcomes of carcinoma of unknown primary trials

), however, the addition of taxanes appears substantially to improve both outcomes. The broad spectrum of activity of the taxanes would predict their efficacy in a heterogeneous condition such as CUP and indeed, the two taxane-based phase II studies with the highest response rates (Hainsworth *et al*, 1997; Briasoulis *et al*, 2000) resulted in median survival times of 13–14 months, almost double those of most other non-taxane regimens. The carboplatin-paclitaxel study (Briasoulis *et al*, 2000) differs from our study and many others in the characteristics of patients included, with 23% of patients diagnosed with peritoneal carcinomatosis (who would be expected to respond well to ‘ovarian cancer’ chemotherapy) and with only 25% of patients recognised as having liver metastases (compared with 65% in our study). When the node only and peritoneal carcinomatosis subsets were removed from the analysis, median survival dropped to 10 months, in keeping with the results of a study of taxane-platinum chemotherapy in a less highly selected group of patients (Greco *et al*, 2001). In the carboplatin-paclitaxel-etoposide study (Hainsworth *et al*, 1997), 24% of patients had node only disease and less than 40% had liver involvement. A randomised phase II comparison of paclitaxel, 5-FU and leucovorin *vs* carboplatin and etoposide (Dowell *et al*, 2001) in 34 patients, 62% of whom had liver metastases, yielded response rates of 19% in both arms, with median survival of less than 9 months, however, the small numbers in each arm again make interpretation difficult.

More important than response rates and median survival data in CUP, where the majority of patients do not respond to chemotherapy, are longer term follow-up data. In our study, 28% of patients survived 1 year, and 10% survived 2 years. This is in keeping with two previous cisplatin-based studies in which 1-year survival is reported as 20–25% (Jadeja *et al*, 1983; Becouarn *et al*, 1989). Three of the taxane-based studies describe more impressive 1- and 2-year survival rates of 42–58% and 20–22% respectively (Hainsworth *et al*, 1997; Greco *et al*, 2000, 2001) and indeed 3-year survival of 14–17% is also quoted (Greco *et al*, 2000, 2001).

While the addition of a taxane to chemotherapy for CUP may well be advantageous, heterogeneity in patient characteristics in phase II studies makes this extremely difficult to demonstrate convincingly at present. This problem is exemplified by the very impressive 15 month median survival quoted for me-FAM (Treat *et al*, 1989), but with only 19 patients of median age 49 years, few of whom had liver metastases, meaningful comparison is impossible.

The heterogeneity of these tumours continues to be the problem, and perhaps the future for the management of cancers of unknown primary lies in improved molecular profiling and better targeted therapy.

## CONCLUSION

MCF appears to be an active regimen in good performance status patients with carcinoma of unknown primary, although in view of the 5-FU-related toxicity observed in this study, a dose of 250 instead of 300 mg m^−2^ day of 5-FU is recommended. The advent of capecitabine may allow the replacement of continuous infusion 5-FU with this oral antimetabolite in due course, removing the potential complications associated with Hickman lines. The role of the taxanes in this heterogeneous disease requires evaluation in a randomised study and our future plans include a comparison of MCF with a taxane based regimen.
